# Prevalence and Clinical Characteristics of Calciphylaxis in Chinese Hemodialysis Patients

**DOI:** 10.3389/fmed.2022.902171

**Published:** 2022-06-10

**Authors:** Yuqiu Liu, Canlin Yang, Xin Yang, Xiaotong Xie, Hong Liu, Liuping Zhang, Jianming Ye, Dongsheng Jiang, Xiaoliang Zhang, Bicheng Liu

**Affiliations:** ^1^Institute of Nephrology, Zhong Da Hospital, School of Medicine, Southeast University, Nanjing, China; ^2^Department of Nephrology, The First People's Hospital of Kunshan, Suzhou, China; ^3^Department of Nephrology, Taizhou People's Hospital, Taizhou, China

**Keywords:** calciphylaxis, clinical characteristics, epidemiological survey, hemodialysis, prevalence, risk factor

## Abstract

**Background:**

Calciphylaxis is a grievous life-threatening vascular disease that commonly affects dialysis population. This is the first epidemiological survey of calciphylaxis initiated in China.

**Methods:**

In the cross-sectional survey, a stratified sampling method was used to select 24 dialysis centers in Jiangsu Province. The participants were all adult patients in each center who had been on hemodialysis for more than 6 months. Calciphylaxis patients were uniformly diagnosed based on characteristic skin lesions and histopathological features.

**Results:**

A total of 3,867 hemodialysis patients (average age of 55.33 ± 13.89 years; 61.81% of males) were included. Forty eight cases were diagnosed with calciphylaxis, and prevalence was 1.24%. Among calciphylaxis patients, 33 cases were male, and the average age and median dialysis duration were 53.85 ± 15.17 years and 84.00 (48.00, 138.75) months, respectively. Skin biopsy was performed in 70.83% of calciphylaxis patients, and positive rate was 64.71%. Meanwhile, the positive rate of bone scintigraphy in the diagnosis of calciphylaxis was 62.5%. The prevalence of hyperparathyroidism in case group was as high as 72.92% with longer duration, and 42.86% had undergone parathyroidectomy. Multivariate analysis indicated that increased BMI, prolonged dialysis duration, warfarin therapy, hyperparathyroidism, diabetes, tumors, low serum albumin and high serum alkaline phosphatase levels were high-risk factors for calciphylaxis.

**Conclusions:**

The prevalence of calciphylaxis in Chinese hemodialysis patients was 1.24% according to regional epidemiological survey, but its actual prevalence would be presumably far beyond present data. It's urgent to improve clinical understanding of calciphylaxis, and multifaceted diagnostic methods should be applied for early screening.

## Introduction

Calciphylaxis, also known as calcific uremic arteriolopathy (CUA), is a destructive vascular disease that mostly occurs in patients with end-stage renal disease (ESRD). Main pathological features are calcification of the subcutaneous adipose tissue and dermal small blood vessel media, along with intimal fibrosis and thrombosis, which lead to tissue ischemic necrosis (1, 2). The clinical manifestations of calciphylaxis are persistent painful skin ulcers and erosions in multiple parts of the body, accompanied by black eschar formation (3–5). And the condition progresses dangerously. The disease is currently difficult to diagnose and treat worldwide.

The epidemiological data of calciphylaxis and its clinical characteristics are of great significance for an in-depth understanding of the disease and exploring diagnosis and treatment plan. It's reported that the estimated annualized incidence of calciphylaxis in maintenance dialysis population worldwide is 1/10,000~35/10,000 (6). The different incidences are possibly related to differences in cognition and diagnostic standards of this disease, and also influenced by race, region, environment, medical condition, and medication habits. However, Chinese research on calciphylaxis remains in its infancy without basic epidemiological data from clinical practice, and only scattered case reports. The present study aims to conduct a multi-center regional epidemiological survey of calciphylaxis among hemodialysis patients in Jiangsu Province, China, to promote the improvement of disease diagnosis and treatment ability.

## Materials and Methods

### Research Objects

In the cross-sectional survey, a stratified sampling method was used to select 24 dialysis centers in four regions of Jiangsu Province. The subjects were all regular hemodialysis patients in each center, who were older than 18-year-old and had been on dialysis for more than 6 months, excluding some mental disorders, intellectual disabilities, and others who couldn't cooperate with the investigation. And written informed consent was obtained from all participants. This research was approved by the Ethics Committee for Clinical Research of Zhongda Hospital Affiliated to Southeast University (Approval number: 2018ZDSYLL100-P01), and registered at the Chinese Clinical Trial Registry (Registration number: ChiCTR1900022248).

### Investigation Methods

The study used a questionnaire that included demographic information, personal history and comorbidities, history of renal and dialysis-related diseases, CKD-MBD and calciphylaxis characteristics, nutrition and microinflammation status. Clinical medical staffs were selected as investigators and trained in advance for the investigation. With informed consent and cooperation, investigators conducted the interview survey on the subjects during the period of hemodialysis, and registered the relevant data on the Internet. For patients with clinically suspected calciphylaxis, histopathological and imaging examinations should be conducted, which would be uniformly interpreted by the calciphylaxis research team of Zhongda Hospital. For patients who had been diagnosed with calciphylaxis, the data at the time of diagnosis were chosen; for others, the last available data were used. In order to ensure the quality of data, additional personnels were arranged to conduct a review, and the proportion of random inspection was 5% to reduce the generation of invalid data. The study dates were from October 2018 to October 2019.

### Statistical Analysis

IBM SPSS Statistics 23 software was used for statistical analysis, and two-sided test *P*- values < 0.05 were considered statistically significant. Measurement data conforming to the normal distribution were expressed as mean ± standard deviation ($\bar{\text{x}}$ ± s), and *t*-test was used for the comparison between groups. Non-normally distributed data were expressed as median [interquartile range (IQR)], and comparisons between groups were performed using the Mann-Whitney *U* rank-sum test. Enumeration data were expressed as number (N) and percentage (%), and unordered categorical variables were compared by Chi-square test or Fisher's exact test, while Mann-Whitney *U* rank-sum test was used for ordered categorical variables. The odds ratio (OR) and 95% confidence interval (CI) were calculated by univariate and multivariate logistic regression analysis to analyze risk factors associated with calciphylaxis.

## Results

### Prevalence of Calciphylaxis in Hemodialysis Population

As of October 31, 2019, a total of 3,867 questionnaires were obtained in four regions of Jiangsu Province, including 1,093 in Nanjing Region, 951 in Southern Jiangsu, 1,156 in Central Jiangsu, and 667 in Northern Jiangsu (Table 1). Among 3,867 hemodialysis patients, 48 cases were diagnosed with calciphylaxis, and the prevalence rate was 1.24%. The lowest prevalence was 0.74% in Southern Jiangsu, while that in Northern Jiangsu was as high as 2.25%. The incidence of calciphylaxis might be related to the level of dialysis management and regional economic development (7). The more developed the area, the lower the prevalence. In addition, 394 (10.32%) of the other 3,819 hemodialysis patients had a variety of manifestations of skin lesions, mainly in lower limbs. They did not meet current diagnostic criteria for calciphylaxis and were excluded from diagnosis based on the unified review by experts. According to the survey data (Supplementary Table 1), 77.04% of hemodialysis patients hadn't heard of calciphylaxis, and the other 9.15% only knew the name without its details, suggesting that the publicity and education of this disease were deficient.

**Table 1:** Regional distribution of hemodialysis population and prevalence of calciphylaxis.

**Area^a^**	**Number of dialysis centers**	**Number of hemodialysis population**				**Per capita GDP (CNY)^b^**
		**Non-calciphylaxis**	**Calciphylaxis**	**Total**	**Prevalence rate**	
Nanjing Region	7	1,078	15	1,093	1.37%	175,600
Southern Jiangsu	6	944	7	951	0.74%	148,400–187,700
Central Jiangsu	6	1,145	11	1,156	0.95%	133,500–146,900
Northern Jiangsu	5	652	15	667	2.25%	74,600–99,900
Total	24	3,819	48	3,867	1.24%	137,300

a *According to the differences in natural characteristics and the economic development level, Jiangsu Province is divided into three sub-regions: The southern area includes Nanjing (provincial capital), Zhenjiang, Changzhou, Wuxi, and Suzhou; the central area includes Nantong, Taizhou, and Yangzhou; and the northern area includes Xuzhou, Lianyungang, Suqian, Huai'an, and Yancheng*.

b *The data comes from the Jiangsu Provincial Bureau of Statistics, which is the per capita gross domestic product (GDP) of cities in Jiangsu Province in 2021*.

### Clinical Features of Different Types of Calciphylaxis

**Figure 1:**
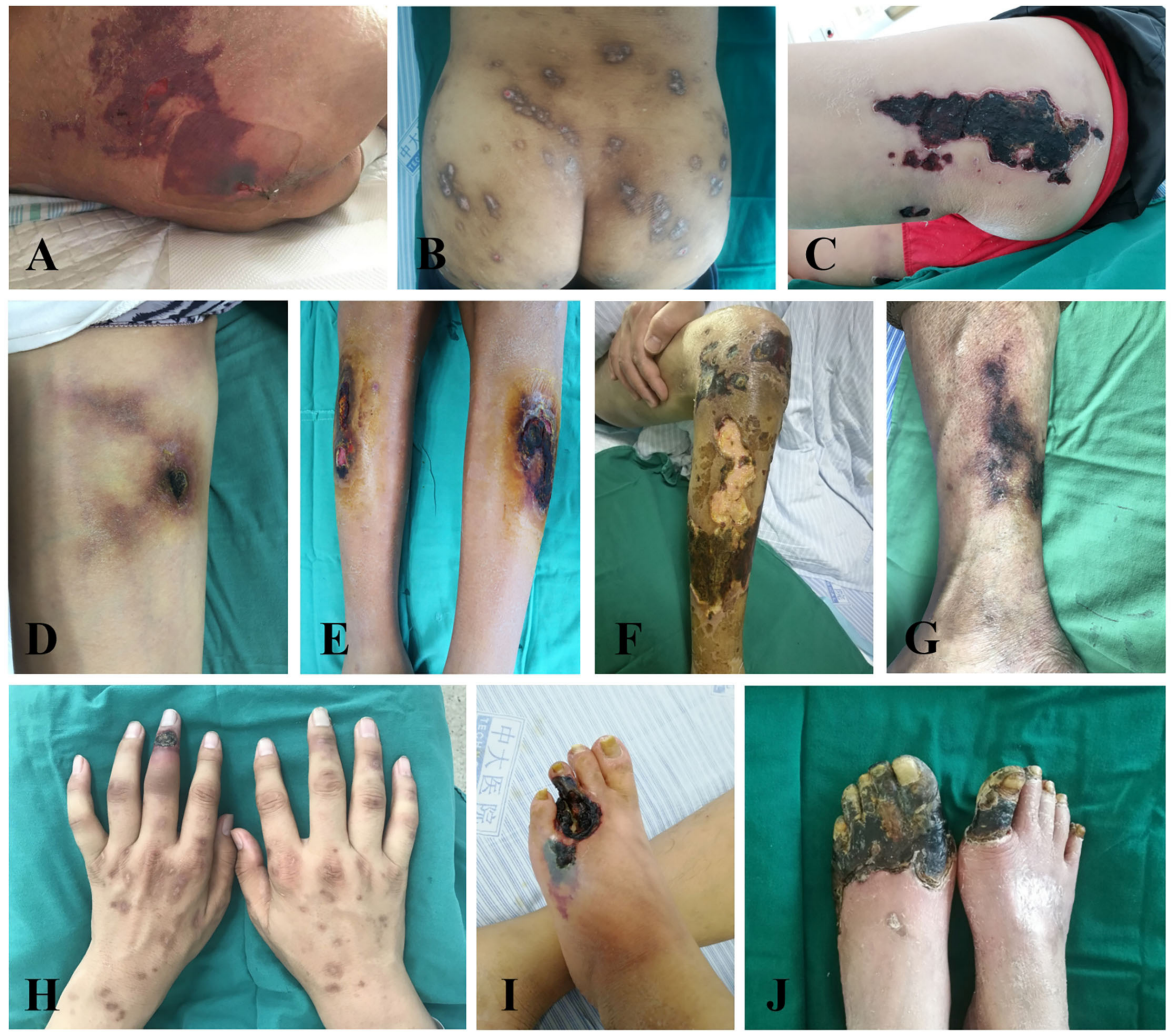
Manifestations of skin lesions with different types of calciphylaxis. According to the affected parts of skin lesions, calciphylaxis can be classified as central and peripheral types. The central calciphylaxis mostly involves fatty central areas such as abdomens, buttocks, and thighs **(A–D)**, while the peripheral type is limited to peripheral parts with a small amount of adipose tissue, such as hands, feet, and penis **(E–J)**. Typical skin lesions of calciphylaxis mainly show livedo reticularis, purpura, sclerotic plaques, necrotic ulcers, and black eschar formation.

**Figure 2:**
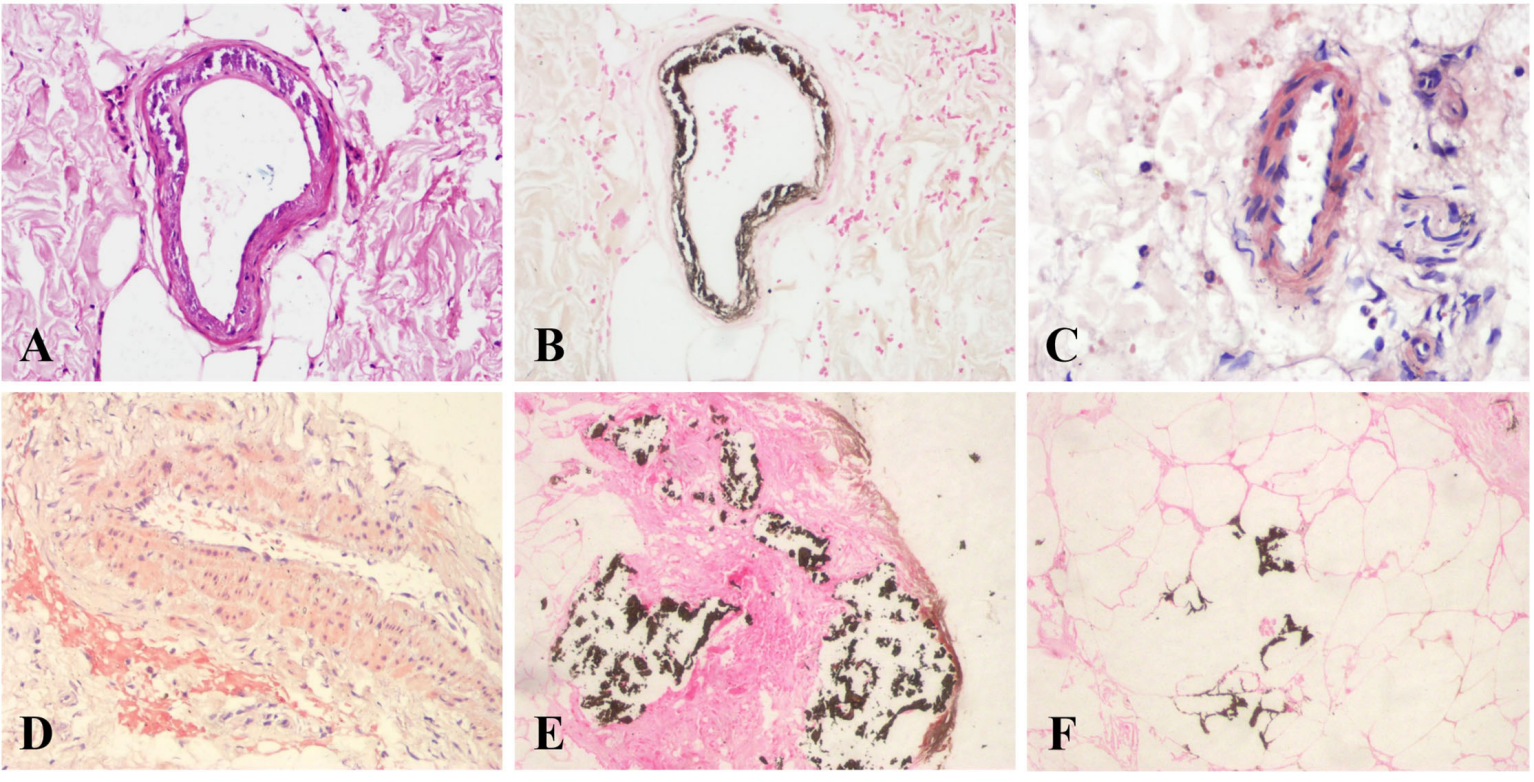
Histopathological features of calciphylaxis. Calciphylaxis skin biopsy specimens show calcification of subcutaneous arteriole media with extensive calcium deposition in extravascular interstitial tissue and fibrous septum of adipose tissue. **(A)** H&E staining, **(B,E,F)** von Kossa staining, and **(C,D)** Alizarin red S staining. Original magnification × 400.

**Figure 3:**
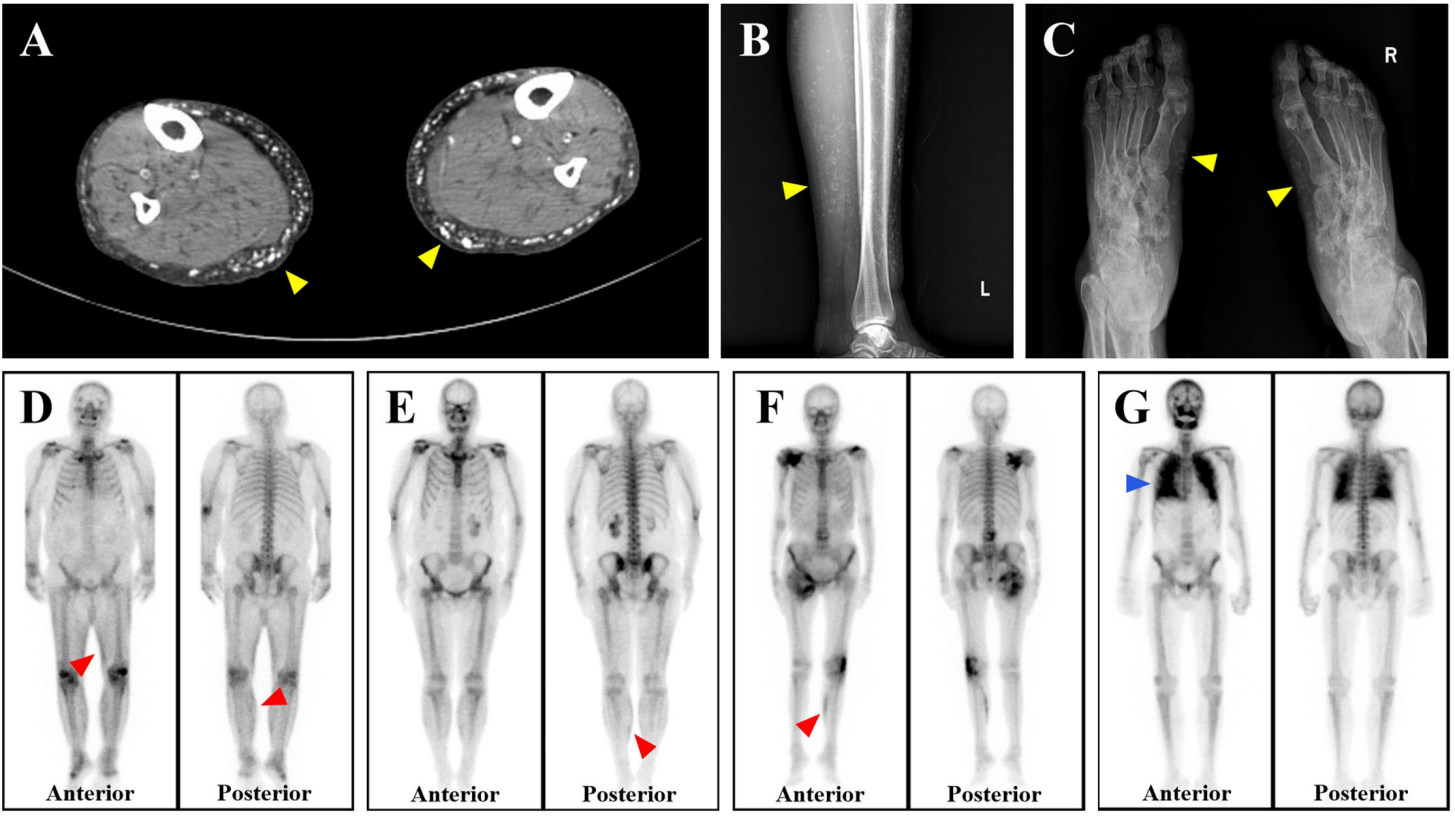
Imaging characteristics of calciphylaxis. **(A–C)** Both CT and X-ray reveal subcutaneous extravascular diffuse calcium deposition of patients with calciphylaxis (yellow arrows, shown as white). **(D–F)** Bone scintigraphy shows the increased uptake of radiotracer by subcutaneous soft tissues of three calciphylaxis patients, especially the continuous linear abnormal radioactive concentration in the lower limbs (red arrows, shown as black). **(G)** Diffuse uptake enhancement is also observed with bone scintigraphy when calciphylaxis involves internal organs such as the lungs (blue arrows).

Among calciphylaxis patients, 33 cases were male, accounting for 68.75%. And the average age and median duration of dialysis were 53.85 ± 15.17 years and 84.00 (48.00, 138.75) months, respectively. As shown in Table 2, 41.67% of the calciphylaxis cases were over 60 years old. The average body mass index (BMI) of calciphylaxis patients was 23.45 ± 4.08 kg/m^2^, of which the highest one was 31.75 kg/m^2^. Overweight people were more susceptible to have this disease, especially the central calciphylaxis. According to the affected part of skin lesions, calciphylaxis could be classified as central type and peripheral type. Figure 1 illustrated the two subtypes. The central type mainly involved the fatty central areas such as breasts, abdomens, buttocks and thighs, while the peripheral type was limited to peripheral parts with a small amount of adipose tissue, such as hands, feet and penis. However, no obvious differences were observed between the two subgroups in terms of gender, age distribution, BMI distribution, and concomitant secondary hyperparathyroidism (SHPT). Histopathological examination of skin biopsy specimens is the gold standard for the diagnosis of calciphylaxis. Skin biopsy was performed in 70.83% of patients with calciphylaxis, especially in peripheral patients whose diagnosis was more dependent on histopathological results. The positive rate of skin biopsy in the diagnosis of calciphylaxis was 64.71%, and the positive rate of subcutaneous arteriolar calcification was higher when samples were taken near central skin lesions (Figure 2). In recent years, bone scintigraphy had been found to be valuable in calciphylaxis diagnosis. We also noticed that calciphylaxis patients tended to have positive results on bone scintigraphy (about 62.5%), mainly characterized by increased uptake or delayed clearance of radioactive tracers in soft tissue, and tracers were mostly distributed in linear or diffuse spots along the subcutaneous surface (Figure 3).

**Table 2:** Comparison of characteristics between central and peripheral calciphylaxis patients.

**Characteristic**	**Total (*N* = 48)**	**Central calciphylaxis (*N* = 20)**	**Peripheral calciphylaxis (*N* = 28)**	***P*-value**
**Gender**
Male	33 (68.75%)	13 (65%)	20 (71.43%)	0.636
Female	15 (31.25%)	7 (35%)	8 (28.57%)	
**Age (years)**
<20	0	0	0	0.588
20–40	9 (18.75%)	5 (25%)	4 (14.29%)	
40–60	19 (39.58%)	7 (35%)	12 (42.86%)	
≥60	20 (41.67%)	8 (40%)	12 (42.86%)	
**BMI(kg/m** ^**2**^ **)**
<18.5	5 (10.42%)	3 (15%)	2 (7.14%)	0.872
18.5–24.0	22 (45.83%)	8 (40%)	14 (50%)	
≥24.0	21 (43.75%)	9 (45%)	12 (42.86%)	
**Duration of dialysis (months)**	84.00 (48.00, 138.75)	91.50 (60.00, 143.75)	69.50 (39.00, 133.75)	0.457
**History of SHPT**
SHPT	35 (72.92%)	15 (75%)	20 (71.43%)	0.784
Duration of SHPT (months)	35.00 (13.00, 72.00)	34.00 (23.00, 72.00)	38.00 (7.00, 67.75)	0.881
Parathyroidectomy	15 (42.86%)	8 (53.33%)	7 (35%)	0.278
Postoperative hypocalcemia	14 (93.33%)	8 (100%)	6 (85.71%)	0.467
**Diagnosis of calciphylaxis**
More than 2 painful and non-treatable skin ulcers	46 (95.83%)	20 (100%)	26 (92.86%)	0.504
Skin biopsy	34 (70.83%)	9 (45%)	25 (89.29%)	0.001
Arteriole calcification on biopsy	22 (64.71%)	8 (88.89%)	14 (56%)	0.173
Bone scintigraphy	32 (66.67%)	7 (35%)	25 (89.29%)	<0.001
Positive result of bone scintigraphy	20 (62.5%)	5 (71.43%)	15 (60%)	0.912

### Comparison of Baseline Characteristics Between Calciphylaxis Cases and Non-calciphylaxis Hemodialysis Patients

A total of 48 calciphylaxis cases and 3,819 cases without calciphylaxis were enrolled in current investigation. Their baseline data were tabulated in Table 3, and there was no significant difference in average age between two groups. The proportion of males in calciphylaxis patients was much higher than that of females, which was consistent with the high ratio of males in the hemodialysis population. Compared with the control group, calciphylaxis patients had longer duration of dialysis [84.00 (48.00, 138.75) vs. 50.00 (24.00, 100.00) months, *P* = 0.002], with the median time of up to 7 years. Although average BMI levels in both groups were within the normal range, the BMI values of patients with calciphylaxis were significantly higher than those in the non-calciphylaxis group (23.45 ± 4.08 vs. 21.80 ± 3.53 kg/m^2^, *P* = 0.008). Hemodialysis patients with diabetes or tumors were more likely to suffer from the disease. 72.92% of calciphylaxis patients were complicated with SHPT. The duration of SHPT in the case group was longer than controls [35.00 (13.00, 72.00) vs. 13.00 (6.00, 36.00) months, *P* = 0.003], and most of them had undergone parathyroidectomy (PTX) (42.86 vs. 23.34%, *P* = 0.008). Albeit only 4 calciphylaxis patients used warfarin therapy, there was still a prominent statistical difference between them and controls (8.33 vs. 0.96%, *P* = 0.002). Laboratory examinations indicated that the levels of white blood cells (WBCs) (7.00 ± 2.24 × 10^9^/L vs. 6.15 ± 1.98 × 10^9^/L, *P* = 0.003), serum albumin (ALB) (36.47 ± 5.06 vs. 40.32 ± 4.74 g/L, *P* < 0.001), serum alkaline phosphatase (ALP) [125.00 (82.00, 208.00) vs. 84.00 (65.00, 115.00) IU/L, *P* < 0.001], serum intact parathyroid hormone (iPTH) [431.30 (129.90, 902.00) vs. 260.00 (120.45, 505.95) pg/mL, *P* = 0.011], and hypersensitive c-reactive protein (hs-CRP) [12.60 (3.73, 30.10) vs. 3.20 (1.00, 7.44) mg/L, *P* < 0.001] were observably different between calciphylaxis and non-calciphylaxis patients.

**Table 3:** Comparison of baseline characteristics between calciphylaxis and non-calciphylaxis hemodialysis patients.

**Characteristic**	**Calciphylaxis patients (*N* = 48)**	**Controls (*N* = 3,819)^b^**	***P*-value**
**Gender**
Male	33 (68.75%)	2,357 (61.72%)	0.319
Female	15 (31.25%)	1,462 (38.28%)	
Age (years)	53.85 ± 15.17	55.35 ± 13.87	0.457
BMI (kg/m^2^)	23.45 ± 4.08	21.80 ± 3.53	0.008
Duration of dialysis (months)	84.00 (48.00, 138.75)	50.00 (24.00, 100.00)	0.002
Kidney transplant	1 (2.08%)	119 (3.25%)	0.964
**Comorbidities**
Hypertension	41 (85.42%)	2,859 (76.59%)	0.150
Diabetes mellitus	18 (37.5%)	701 (19.07%)	0.001
Coronary heart disease	8 (16.67%)	369 (10.04%)	0.204
Atrial fibrillation	6 (12.5%)	186 (5.13%)	0.051
Stroke	8 (16.67%)	305 (8.32%)	0.071
Hepatitis	8 (16.67%)	492 (13.27%)	0.491
Tumor	4 (8.33%)	32 (0.87%)	0.001
**History of SHPT**
SHPT	35 (72.92%)	1,364 (38.03%)	<0.001
Duration of SHPT (months)	35.00 (13.00, 72.00)	13.00 (6.00, 36.00)	0.003
Parathyroidectomy	15 (42.86%)	302 (23.34%)	0.008
Postoperative hypocalcemia	14 (93.33%)	225 (76.27%)	0.223
**Medication history**
Immunosuppressive therapy	11 (22.92%)	555 (15%)	0.128
Warfarin therapy	4 (8.33%)	30 (0.96%)	0.002
**Laboratory examination**
Hemoglobin (g/L)	108.69 ± 21.21	106.77 ± 19.75	0.503
White blood cell ( ×10^9^/L)	7.00 ± 2.24	6.15 ± 1.98	0.003
Platelet ( ×10^9^/L)	179.51 ± 67.39	164.61 ± 59.20	0.087
Serum calcium (mmol/L)	2.34 ± 0.29	2.30 ± 0.41	0.535
Corrected serum calcium^a^ (mmol/L)	2.41 ± 0.29	2.30 ± 0.42	0.072
Serum phosphate (mmol/L)	1.92 ± 0.65	1.89 ± 0.68	0.757
Serum albumin (g/L)	36.47 ± 5.06	40.32 ± 4.74	<0.001
ALP (IU/L)	125.00 (82.00, 208.00)	84.00 (65.00, 115.00)	<0.001
ALT (IU/L)	16.00 (9.25, 21.50)	12.00 (8.00, 18.00)	0.167
AST (IU/L)	16.00 (12.25, 21.00)	14.00 (11.00, 19.00)	0.125
Triglycerides (mmol/L)	1.38 (0.91,2.60)	1.50 (1.00,2.40)	0.574
Total cholesterol (mmol/L)	3.74 (3.14, 4.35)	3.60 (3.04, 4.40)	0.767
iPTH (pg/mL)	431.30 (129.90, 902.00)	260.00 (120.45, 505.95)	0.011
Plasma glucose (fasting) (mmol/L)	5.56 (4.76, 6.83)	5.40 (4.67, 7.02)	0.736
Glycated hemoglobin (%)	7.28 ± 1.60	6.52 ± 1.76	0.090
INR	1.11 ± 0.12	1.07 ± 0.18	0.082
Ferritin (ug/L)	103.60 (38.40, 403.20)	194.30 (69.10, 497.20)	0.091
TnI (ng/mL)	0.034 (0.012, 0.058)	0.030 (0.010, 0.070)	0.938
BNP (pg/mL)	342.00 (95.00, 1060.00)	744.45 (204.50, 2220.00)	0.137
hs-CRP (mg/L)	12.60 (3.73, 30.10)	3.20 (1.00, 7.44)	<0.001

a *Corrected serum calcium: The serum calcium level was corrected based on the albumin content, and the formula was: corrected serum Ca concentration (mg/dL) = measured Ca concentration (mg/dL) + 0.8 × [4.0 - measured serum albumin concentration (g/dL)]*.

b *Percentages of some items might not be equal to the ratio of the numbers to the total number of groups due to partial incomplete data*.

### Risk Factors Associated With the Development of Calciphylaxis

**Figure 4:**
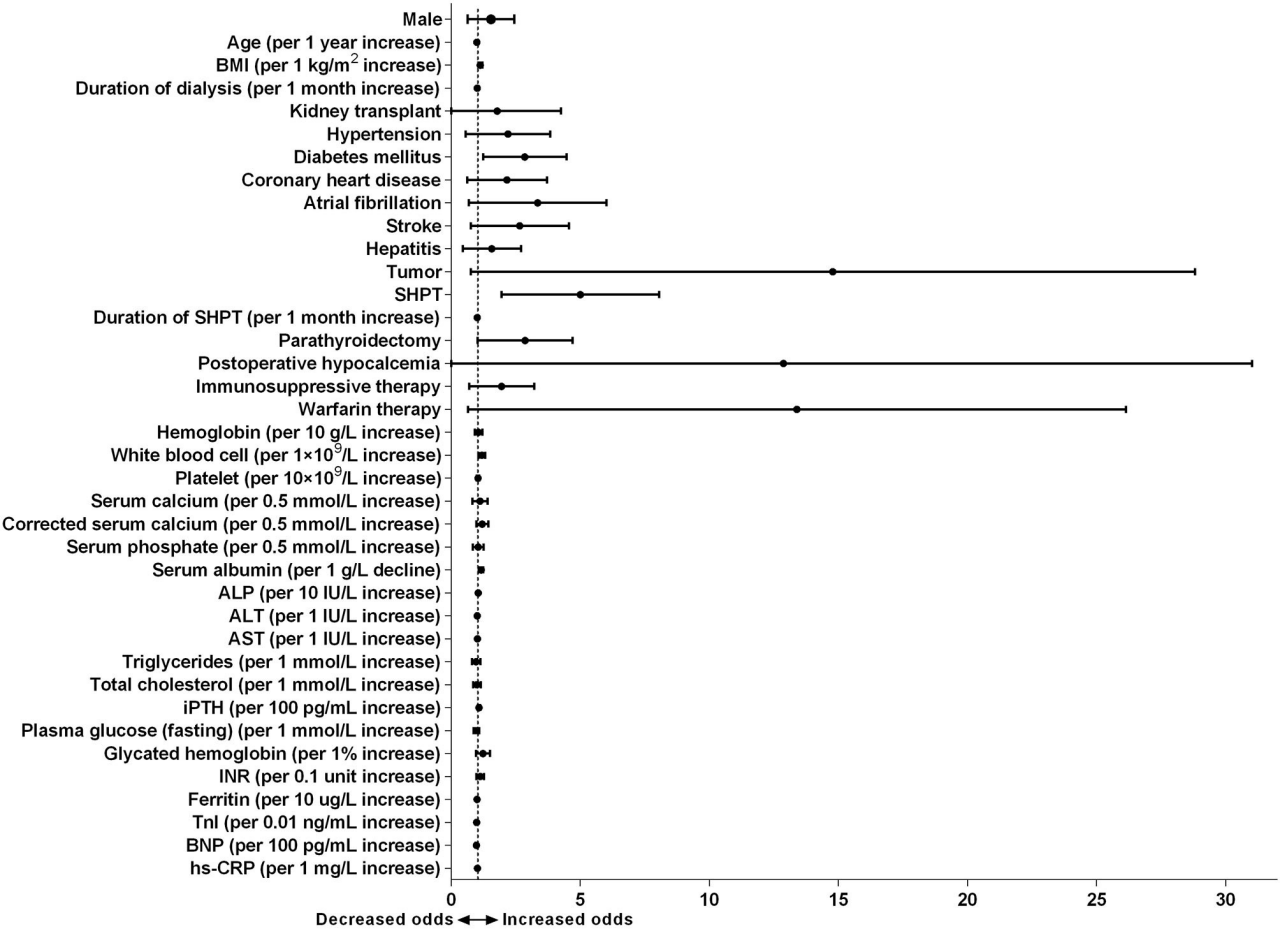
Forest plot of odds ratio of risk factors for calciphylaxis development based on univariate logistic regression analyses. Univariate logistic regression model shows odds ratio (OR) of calciphylaxis development by patient characteristics at the time of diagnosis. Filled circles denote point estimate of OR and error bars represent 95% confidence interval (CI).

As summarized in Figure 4, the forest plot showed the results of univariate logistic regression analysis to tell from risk factors related to the development of calciphylaxis. Neither gender nor age showed a correlation with calciphylaxis. Whereas, each 1 kg/m^2^ increase in BMI was associated with an increased risk of the disease (OR 1.121, 95% CI 1.046–1.201, *P* = 0.001) (Supplementary Table 2). There was an arresting correlation between increased duration of dialysis and calciphylaxis (OR 1.006, 95% CI 1.002–1.010, *P* = 0.002), but no effect on it was observed in immunosuppressive therapy and other treatment measures such as kidney transplantation. In particular, warfarin therapy significantly increased the risk of calciphylaxis (OR 9.352, 95% CI 3.160–27.672, *P* < 0.001). In term of comorbidities, univariate analysis revealed that diabetes mellitus, atrial fibrillation, stroke and tumors were all closely associated with calciphylaxis. SHPT was one of the vital complications of ESRD, which might be involved in the occurrence and development of calciphylaxis (OR 4.388, 95% CI 2.313–8.323, *P* < 0.001). Each additional month of SHPT duration (OR 1.008, 95% CI 1.002–1.014, *P* = 0.007) and PTX (OR 2.464, 95% CI 1.246–4.871, *P* = 0.010) would further increase the risk of this disease. According to blood test results, each 1 × 10^9^/L increase in WBCs level (OR 1.178, 95% CI 1.057–1.314, *P* = 0.003), each 10 IU/L increase in ALP level (OR 1.046, 95% CI 1.030–1.063, *P* < 0.001), each 100 pg/mL increase in iPTH level (OR 1.080, 95% CI 1.042–1.120, *P* < 0.001), each 1 mg/L increase in hs-CRP level (OR 1.018, 95% CI 1.010–1.025, *P* < 0.001), and 1 g/L decrease in ALB level (OR 1.166, 95% CI 1.105–1.230, *P* < 0.001) at the time of diagnosis were markedly correlated with calciphylaxis.

The factors identified as significant in the univariate model were incorporated into the multivariate regression model. Since SHPT was correlated with duration of SHPT and PTX, they could not be contained simultaneously in the multivariate analysis. Only SHPT was included as a covariate in the multivariate statistics. The results listed in Table 4 demonstrated that increased BMI, prolonged dialysis duration, concomitant diabetes mellitus or tumors, concurrent SHPT, warfarin therapy, decreased level of ALB and elevated level of ALP remained significantly associated with calciphylaxis, which were independent high-risk factors for this disease. Among them, warfarin received special attention as one of the classic risk factors of calciphylaxis, which increased its risk by 38.677-fold.

**Table 4:** Multivariate logistic regression analysis to determine risk factors of calciphylaxis in hemodialysis patients.

**Characteristic**	***P*-value**	**OR (95%CI)**
BMI (per 1 kg/m^2^ increase)	0.003	1.154 (1.049–1.270)
Duration of dialysis (per 1 month increase)	0.002	1.009 (1.003–1.015)
Diabetes mellitus	0.026	2.798 (1.128–6.945)
Tumor	0.021	7.186 (1.343–38.442)
SHPT^a^	0.005	3.460 (1.448–8.272)
Warfarin therapy	0.004	38.677 (3.249–460.451)
Serum albumin (per 1 g/L decline)	<0.001	1.205 (1.113–1.305)
ALP (per 10 IU/L increase)	0.023	1.036 (1.005–1.069)

a *Since SHPT was correlated with the duration of SHPT and parathyroidectomy, only SHPT was included as a covariate in the multivariate analysis*.

## Discussion

This is the first epidemiological data on calciphylaxis based on Chinese population, involving 24 hemodialysis centers. The preliminary analysis shows that the prevalence of calciphylaxis in hemodialysis patients is 1.24%. Some patients with “atypical (atypical skin lesions)” calciphylaxis have not been contained in the statistics, hence the actual prevalence is presumably far beyond the estimate. A nationwide survey of hemodialysis centers conducted in Japan observed that <10% of nephrologists mastered the standardized diagnosis and treatment methods for calciphylaxis (8). As a disease with such a high disability and mortality, it should attract the attention of relevant specialists.

Calciphylaxis is considered to be a multifactorial disease, and analyzing its risk factors can provide important clues for disease diagnosis. The calciphylaxis patients in our survey were mainly male, but the effect of gender difference on the incidence of this disease was not observed, which was considered to be related to the high proportion of male in the hemodialysis population. Obesity has been found to be one of the risk factors for calciphylaxis (9). Though the average BMI of patients in present study did not meet the criteria for obesity, the level of case group was still significantly higher than that of control group, which was in accordance with previous studies (10). Central calciphylaxis tends to occur in fatty areas, therefore obesity presumably increase the risk of developing this subtype (11). In future, it is necessary to expand the sample size of calciphylaxis patients to further determine the appropriate BMI cut-off value for guiding clinical practice. Calciphylaxis is more common in dialysis population, and the disease risk increases gradually with the extension of dialysis duration. In the meantime, this investigation also found that the prevalence of calciphylaxis was probably related to the local economic development level. Since area with low economic level and hospital assistance have an increased risk of this disease and should be well trained for properly diagnosis and prevention. Favorable dialysis management can reduce the incidence of the disease to a certain extent. Dialysis can be intensified by prolonging dialysis duration, increasing dialysis frequency and using hemofiltration, but intensification beyond the goal of dialysis adequacy is not recommended (5, 12).

SHPT is a crucial risk factor for calciphylaxis. The prevalence and duration of SHPT in patients with calciphylaxis were higher than those in the control group in current survey, and the median iPTH level at the time of diagnosis was 431.30 pg/mL. This suggests that SHPT is poorly controlled in Chinese calciphylaxis patients, which may be related to the low use rate of cinacalcet. The EVOLVE trial reported that cinacalcet appeared to reduce the incidence of calciphylaxis in hemodialysis patients with moderate to severe SHPT (13). And cinacalcet combined with sodium thiosulfate to treat calciphylaxis could also improve the outcome of this devastating disease (14). Moreover, 42.86% of calciphylaxis patients with SHPT had undergone PTX at the time of diagnosis, and their median iPTH level decreased from 734.65 to 306.0 pg/mL compared with the non-PTX group. In general, calciphylaxis patients accompanied by observably elevated iPTH should be treated with cinacalcet or PTX, but it is necessary to avoid the rapid decline and excessive inhibition of iPTH in a short period of time, which presumably affect the bone turnover state, thereby inducing or aggravating calciphylaxis (15, 16). Patients with diabetes mellitus and cancer had a preference for calciphylaxis. Warfarin, as an anticoagulant commonly used in clinic, is a well-recognized potential precipitating factor of calciphylaxis (17, 18). In this epidemiological investigation, there was found that it memorably increased the risk of calciphylaxis although only 8.33% of patients had a history of warfarin therapy. Calciphylaxis skin lesions were prone to secondary infection (19), and the levels of WBCs and hs-CRP were significantly elevated. After multivariate analysis, low ALB level and high ALP level were identified as independent risk factors for calciphylaxis, which was in keeping with the results of a matched case-control study conducted by us previously (20). As another important risk factor for calciphylaxis, several researches have discovered that a significant decrease in albumin level in patients with typical malnutrition (21, 22), which requires strengthening supportive treatment, such as improving nutrition, supplementing albumin and correcting anemia.

The currently accepted diagnostic criteria of calciphylaxis are mainly based on high-risk factors, characteristic skin lesions and histopathological features (23, 24), so it's hard to recognize the early stages of the disease (5). Early diagnosis of calciphylaxis patients is a key measure to reduce their high disability rate and high mortality. Blood examinations can only provide early warning information for preliminary screening of calciphylaxis (25). Notwithstanding tissue biopsy is the gold standard, sometimes a repeated biopsy is necessary to obtain the pathological diagnosis, which will delay the treatment and may cause new skin lesions (6, 26, 27). Hence, skin biopsy and surgical debridement should be sufficient to obtain a diagnosis and reduce morbidity associated with surgery, especially in acral sites such as the penis (28). A few case reports had described the high sensitivity and specificity of bone scintigraphy in the diagnosis of calciphylaxis (29, 30), which had been confirmed in our investigation. When calciphylaxis occurs in uremic patients, there is “new bone formation” in the soft tissues (31), and Tc-99m MDP bone scan can detect osteoblast activity through hydroxyapatite crystals chemisorbed into the newly formed bone (32). Hence, as a non-invasive large-scale detection method, bone scintigraphy is probably of great significance in early diagnosis before the appearance of ulcerative lesions, and it can also be used as a means of curative effect monitoring. Especially in our investigation, we noticed that peripheral calciphylaxis skin lesions were usually atypical (33), which were easily confused with a wide variety of other diseases, including diabetic ulcers, atherosclerotic vascular diseases and thromboangiitis obliterans, etc. (34). In consequence, the diagnosis of patients with peripheral calciphylaxis depends more on auxiliary examinations. It suggests that bone scan technology could be applied in clinical work in the future to screen these high-risk patients to get clues for further examinations such as skin biopsy to make an early diagnosis of calciphylaxis.

China has a huge number of ESRD patients receiving dialysis treatment, among whom there are quite a few potential calciphylaxis patients. Whereas, Chinese research on calciphylaxis is so insufficient that it's urgent to improve clinical understanding of it. Applying multifaceted diagnostic methods for early screening and strengthening the prevention of the disease are extremely crucial to improve the overall ability of calciphylaxis diagnosis and treatment.

## Data Availability Statement

The raw data supporting the conclusions of this article will be made available by the authors, without undue reservation.

## Ethics Statement

The studies involving human participants were reviewed and approved by the Ethics Committee for Clinical Research of Zhongda Hospital Affiliated to Southeast University (Approval Number: 2018ZDSYLL100-P01). The patients/participants provided their written informed consent to participate in this study. Written informed consent was obtained from the individual(s) for the publication of any potentially identifiable images or data included in this article.

## Author Contributions

Data acquisition: YL, CY, XY, XX, and LZ. Drafting and editing manuscript: YL and XZ. Administrative, technical, or material support: HL, JY, DJ, XZ, and BL. Study supervision and mentorship: XZ and BL. Study conception, design, data analysis, and interpretation: all authors. All authors contributed important intellectual content during manuscript drafting or revision, accepts personal accountability for the author's own contributions, and agrees to ensure that questions about the accuracy or integrity of any portion of the work are appropriately investigated and resolved. All authors contributed to the article and approved the submitted version.

## Funding

This work was funded by the National Natural Science Foundation of China (Grant Nos. 81570612 and 81870497), the Jiangsu Provincial Key Research and Development Program (Grant No. BE2021737), and the Nanjing Health Science and Technology Development Project (Grant No. YKK20237).

## Conflict of Interest

The authors declare that the research was conducted in the absence of any commercial or financial relationships that could be construed as a potential conflict of interest.

## Publisher's Note

All claims expressed in this article are solely those of the authors and do not necessarily represent those of their affiliated organizations, or those of the publisher, the editors and the reviewers. Any product that may be evaluated in this article, or claim that may be made by its manufacturer, is not guaranteed or endorsed by the publisher.

## Abbreviations

ALP: Serum alkaline phosphatase
ALT: Alanine aminotransferase
AST: Aspartate aminotransferase
BMI: Body mass index
BNP: Brain natriuretic peptide
CI: Confidence interval
hs-CRP: Hypersensitive c-reactive protein
INR: International normalized ratio
iPTH: Serum intact parathyroid hormone
OR: Odds ratio
SHPT: Secondary hyperparathyroidism
TnI: Troponin I.
