# Supplementary material for: Prevalence and Clinical Characteristics of Calciphylaxis in Chinese Hemodialysis Patients
Source: Front Med (Lausanne). 2022 Jun 10;9:902171. doi: 10.3389/fmed.2022.902171 (PMC9226545; doi:10.3389/fmed.2022.902171)
Supplement: Supplementary file 1 [file Data_Sheet_1.docx]

***Supplementary Material***

**Supplementary Table 1. Awareness rate of calciphylaxis in hemodialysis population**

| **Degree of understanding** | **Non-calciphylaxis** | **Calciphylaxis** | **Total** | **Percentage** |
| --- | --- | --- | --- | --- |
| Not heard | 2945 | 34 | 2979 | 77.04% |
| Only heard of the name | 345 | 9 | 354 | 9.15% |
| A little understanding | 366 | 2 | 368 | 9.52% |
| Basic understanding | 127 | 2 | 129 | 3.34% |
| Detailed understanding | 12 | 1 | 13 | 0.34% |
| No data | 24 | 0 | 24 | 0.62% |
| Total | 3819 | 48 | 3867 | 100% |

**Supplementary Table 2. Clinical characteristics related to calciphylaxis in hemodialysis patients assessed by univariate logistic regression analyses**

| **Characteristic** | ***P*-value** | **OR (95%CI)** |
| --- | --- | --- |
| **Male** | 0.321 | 1.365 (0.739-2.521) |
| **Age (per 1 year increase)** | 0.457 | 0.992 (0.972-1.013) |
| **BMI (per 1 kg/m^2^ increase)** | 0.001 | 1.121 (1.046-1.201) |
| **Duration of dialysis (per 1 month increase)** | 0.002 | 1.006 (1.002-1.010) |
| **Kidney transplant** | 0.652 | 0.632 (0.087-4.622) |
| ***Comorbidities*** |  | |
| Hypertension | 0.156 | 1.791 (0.800-4.005) |
| Diabetes mellitus | 0.002 | 2.546 (1.411-4.593) |
| Coronary heart disease | 0.136 | 1.791 (0.832-3.856) |
| Atrial fibrillation | 0.028 | 2.642 (1.109-6.294) |
| Stroke | 0.044 | 2.203 (1.022-4.748) |
| Hepatitis | 0.492 | 1.308 (0.609-2.810) |
| Tumor | ＜0.001 | 10.347 (3.510-30.501) |
| ***History of SHPT*** |  | |
| SHPT | ＜0.001 | 4.388 (2.313-8.323) |
| Duration of SHPT (per 1 month increase) | 0.007 | 1.008 (1.002-1.014) |
| Parathyroidectomy | 0.010 | 2.464 (1.246-4.871) |
| Postoperative hypocalcemia | 0.159 | 4.356 (0.563-33.712) |
| ***Medication history*** |  | |
| Immunosuppressive therapy | 0.132 | 1.685 (0.854-3.322) |
| Warfarin therapy | ＜0.001 | 9.352 (3.160-27.672) |
| ***Laboratory examination*** |  | |
| Hemoglobin (per 10 g/L increase) | 0.503 | 1.051 (0.909-1.214) |
| White blood cell (per 1×10^9^/L increase) | 0.003 | 1.178 (1.057-1.314) |
| Platelet (per 10×10^9^/L increase) | 0.088 | 1.040 (0.994-1.087) |
| Serum calcium (per 0.5 mmol/L increase) | 0.535 | 1.089 (0.833-1.423) |
| Corrected serum calcium ^a^ (per 0.5 mmol/L increase) | 0.082 | 1.186 (0.978-1.438) |
| Serum phosphate (per 0.5 mmol/L increase) | 0.756 | 1.032 (0.844-1.263) |
| Serum albumin (per 1 g/L decline) | ＜0.001 | 1.166 (1.105-1.230) |
| ALP (per 10 IU/L increase) | ＜0.001 | 1.046 (1.030-1.063) |
| ALT (per 1 IU/L increase) | 0.572 | 1.006 (0.986-1.025) |
| AST (per 1 IU/L increase) | 0.090 | 1.013 (0.998-1.027) |
| Triglycerides (per 1 mmol/L increase) | 0.608 | 0.955 (0.803-1.137) |
| Total cholesterol (per 1 mmol/L increase) | 0.862 | 0.987 (0.854-1.142) |
| iPTH (per 100 pg/mL increase) | ＜0.001 | 1.080 (1.042-1.120) |
| Plasma glucose (fasting) (per 1 mmol/L increase) | 0.590 | 0.970 (0.868-1.084) |
| Glycated hemoglobin (per 1% increase) | 0.095 | 1.211 (0.967-1.516) |
| INR (per 0.1 unit increase) | 0.090 | 1.116 (0.983-1.268) |
| Ferritin (per 10 ug/L increase) | 0.526 | 0.998 (0.990-1.005) |
| TnI (per 0.01 ng/mL increase) | 0.423 | 0.988 (0.958-1.018) |
| BNP (per 100 pg/mL increase) | 0.212 | 0.974 (0.936-1.015) |
| hs-CRP (per 1 mg/L increase) | ＜0.001 | 1.018 (1.010-1.025) |

^a^ Corrected serum calcium: The serum calcium level was corrected based on the albumin content, and the formula was: corrected serum Ca concentration (mg/dL) = measured Ca concentration (mg/dL) + 0.8 × [4.0 － measured serum albumin concentration (g/dL) ].
